# Thermal acclimation and habitat-dependent differences in temperature robustness of a crustacean motor circuit

**DOI:** 10.3389/fncel.2023.1263591

**Published:** 2023-10-18

**Authors:** Wolfgang Stein, Gabriela Torres, Luis Giménez, Noé Espinosa-Novo, Jan Phillipp Geißel, Andrés Vidal-Gadea, Steffen Harzsch

**Affiliations:** ^1^School of Biological Sciences, Illinois State University, Normal, IL, United States; ^2^Stiftung Alfried Krupp Kolleg Greifswald, Greifswald, Germany; ^3^Alfred-Wegener-Institut, Helmholtz-Zentrum für Polar- und Meeresforschung, Biologische Anstalt Helgoland, Helgoland, Germany; ^4^School of Ocean Sciences, Bangor University, Bangor, United Kingdom; ^5^Department of Cytology and Evolutionary Biology, Zoological Institute and Museum, University of Greifswald, Greifswald, Germany

**Keywords:** stomatogastric ganglion, central pattern generation, climate change, degeneracy, acclimatization, phase constancy, robustness

## Abstract

**Introduction:**

At the cellular level, acute temperature changes alter ionic conductances, ion channel kinetics, and the activity of entire neuronal circuits. This can result in severe consequences for neural function, animal behavior and survival. In poikilothermic animals, and particularly in aquatic species whose core temperature equals the surrounding water temperature, neurons experience rather rapid and wide-ranging temperature fluctuations. Recent work on pattern generating neural circuits in the crustacean stomatogastric nervous system have demonstrated that neuronal circuits can exhibit an intrinsic robustness to temperature fluctuations. However, considering the increased warming of the oceans and recurring heatwaves due to climate change, the question arises whether this intrinsic robustness can acclimate to changing environmental conditions, and whether it differs between species and ocean habitats.

**Methods:**

We address these questions using the pyloric pattern generating circuits in the stomatogastric nervous system of two crab species, *Hemigrapsus sanguineus* and *Carcinus maenas* that have seen a worldwide expansion in recent decades.

**Results and discussion:**

Consistent with their history as invasive species, we find that pyloric activity showed a broad temperature robustness (>30°C). Moreover, the temperature-robust range was dependent on habitat temperature in both species. Warm-acclimating animals shifted the critical temperature at which circuit activity breaks down to higher temperatures. This came at the cost of robustness against cold stimuli in *H. sanguineus*, but not in *C. maenas*. Comparing the temperature responses of *C. maenas* from a cold latitude (the North Sea) to those from a warm latitude (Spain) demonstrated that similar shifts in robustness occurred in natural environments. Our results thus demonstrate that neuronal temperature robustness correlates with, and responds to, environmental temperature conditions, potentially preparing animals for changing ecological conditions and shifting habitats.

## Introduction

Temperature is a critical environmental factor that profoundly influences the physiological processes and behaviors of organisms (Hofmann and Todgham, 2010). The nervous system is particularly affected by temperature changes since these can significantly disrupt cellular and circuit activity (Robertson and Money, 2012; Harding et al., 2019; Marder and Rue, 2021; Robertson et al., 2023) with far-reaching consequences for animal survival and performance. Recent research focusing on understanding the effects of temperature on neural circuits in poikilothermic invertebrates, animals that are especially susceptible to climate change-induced global warming and the associated severe weather events (Reid et al., 2009; Somero, 2010; Somero, 2012; Mann et al., 2018; Oliver et al., 2018), has revealed that some neural circuits display automatic robustness to acute temperature perturbations. Studies on the pyloric central pattern generating circuits in the crustacean stomatogastric ganglion (STG) have demonstrated the existence of a surprisingly large robustness to temperature fluctuations that maintains normal activity patterns over a broad range of temperatures (7–27°C) (Tang et al., 2010, 2012; Soofi et al., 2014). This robustness is either intrinsic to the circuit and arises from the emergent properties of cellular and synaptic ionic conductances (Alonso and Marder, 2020) or is a consequence of homeostatic compensatory mechanisms. The pyloric rhythm regulates the contraction and relaxation of muscles involved in food digestion and is continuously active throughout the life span of the animal (Stein, 2017), but its responses to temperature changes have only been studied in two related crab species (*Cancer borealis* and *Cancer pagurus*) that live in the epipelagic and mesopelagic zones (up to a depth of 750 m). With ongoing global ocean warming and recurring heatwaves with rapidly changing temperatures affecting intertidal and pelagic habitats of marine crustaceans, it now becomes crucial to explore the limits of temperature robustness, and to investigate potential regulatory mechanisms, species-specific differences, and adaptation to altered environmental conditions.

This study examines the temperature responses of the pyloric pattern generating circuits in two crab species, *Hemigrapsus sanguineus* and *Carcinus maenas*. These species live in the intertidal zone (Crothers, 1968; Klassen, 2012; Epifanio, 2013) and are thus exposed to the most extreme ocean temperature changes. They are originally native to the temperate waters of Asia and Europe, respectively (Klassen and Locke, 2007; Klassen, 2012), but have experienced a worldwide expansion in recent decades causing a significant threat to invaded ecosystems. The two species inhabit overlapping niches in the North Sea where they compete for food and shelter (Dauvin and Dufossé, 2011; Geburzi et al., 2018), providing a unique opportunity to study their responses to temperature fluctuations in a common habitat. Our primary research question focused on determining the range of temperatures that enables a continuous and regular pyloric rhythm, thus providing insights into the temperature robustness of the neural circuitry in these species. By subjecting isolated stomatogastric nervous systems to acute temperature changes, we examined temperature effects on the pyloric rhythm and assessed the rhythm’s overall robustness. We also explored changes in the structure of the rhythm, i.e., the phase relationships of the pyloric neurons with temperature. Phase constancy, the maintenance of stable phase relationships within the pattern, has been proposed as a critical feature of rhythmic activity in the pyloric rhythm (Hooper, 1997; Bucher et al., 2005; Mouser et al., 2008), although its underlying mechanisms are not always known. Finally, we explored the relationship between habitat temperature and temperature robustness in both species by warm-acclimating animals for 2 months and comparing their temperature responses to non-acclimated individuals and animals that experienced warm-acclimatization in their natural habitat.

Understanding the temperature robustness of neuronal circuits in crab species with high invasive potential is of ecological and evolutionary significance, as it provides insights into their capacity to withstand changing environmental conditions and shifts in their habitat.

## Materials and methods

### Animals

Adult *Carcinus maenas* were collected in Helgoland, Germany, and Vigo, Spain. Adult *Hemigrapsus sanguineus* were collected in Helgoland, Germany. Animals were housed individually and transported to the University of Greifswald, where they were kept at their respective habitat temperatures for at least 2 months before being used for experiments. While on Helgoland, crabs were fed frozen shrimp (*Crangon crangon*) twice a week. In Greifswald, crabs were kept in air-bubbled artificial seawater (Sera reef salt, Heinsberg, Germany) that was exchanged every 10 days. Animals were fed frozen shrimp (*Litopenaeus vannamei*) once a week. Habitat temperatures were 8°C (*C. maenas* from Helgoland), 15°C (*C. maenas* from Vigo), and 15°C (*H. sanguineus* from Helgoland). A subset of the *C. maenas* crabs collected in Helgoland was acclimated to 15°C for at least 2 months. A subset of the *H. sanguineus* crabs was acclimated to 25°C for at least 2 months. The research presented in this paper complies with national (Germany) and international laws (guidelines from the directives 2010/63/EU of the European parliament and of the Council of 22nd September 2010) on the protection of animals used for scientific purposes.

### Solutions

*Carcinus maenas* saline solution was composed of 410 mM NaCl, 11 mM KCl, 11 mM CaCl_2_, 21 mM MgCl2, 30 mM NaHCO_3_, 11 mM Tris, 5.1 mM maleic acid; pH 7.4–7.6 (Siebers et al., 1972). *H. sanguineus* saline solution was composed of 410 mM NaCl, 8.3 mM KCl, 10.3 mM CaCl_2_, 52.3 mM MgCl_2_, 2 mM NaHCO_3_, 33.4 mM NaSO_4_, 0.84 mM KBr, 0.410 mM H_3_BO_3_, 0.090 mM SrCl_2_, 0.069 mM NaF; pH 7.4–7.6.

### Dissection

Animals were anesthetized on ice for at least 30 min. The stomatogastric nervous system was dissected following standard procedures as described previously (Gutierrez and Grashow, 2009; Städele et al., 2015) and pinned out in a silicone-lined petri dish (Sylgard 184, Dow Corning).

### Electrophysiological recordings

**Figure 1:**
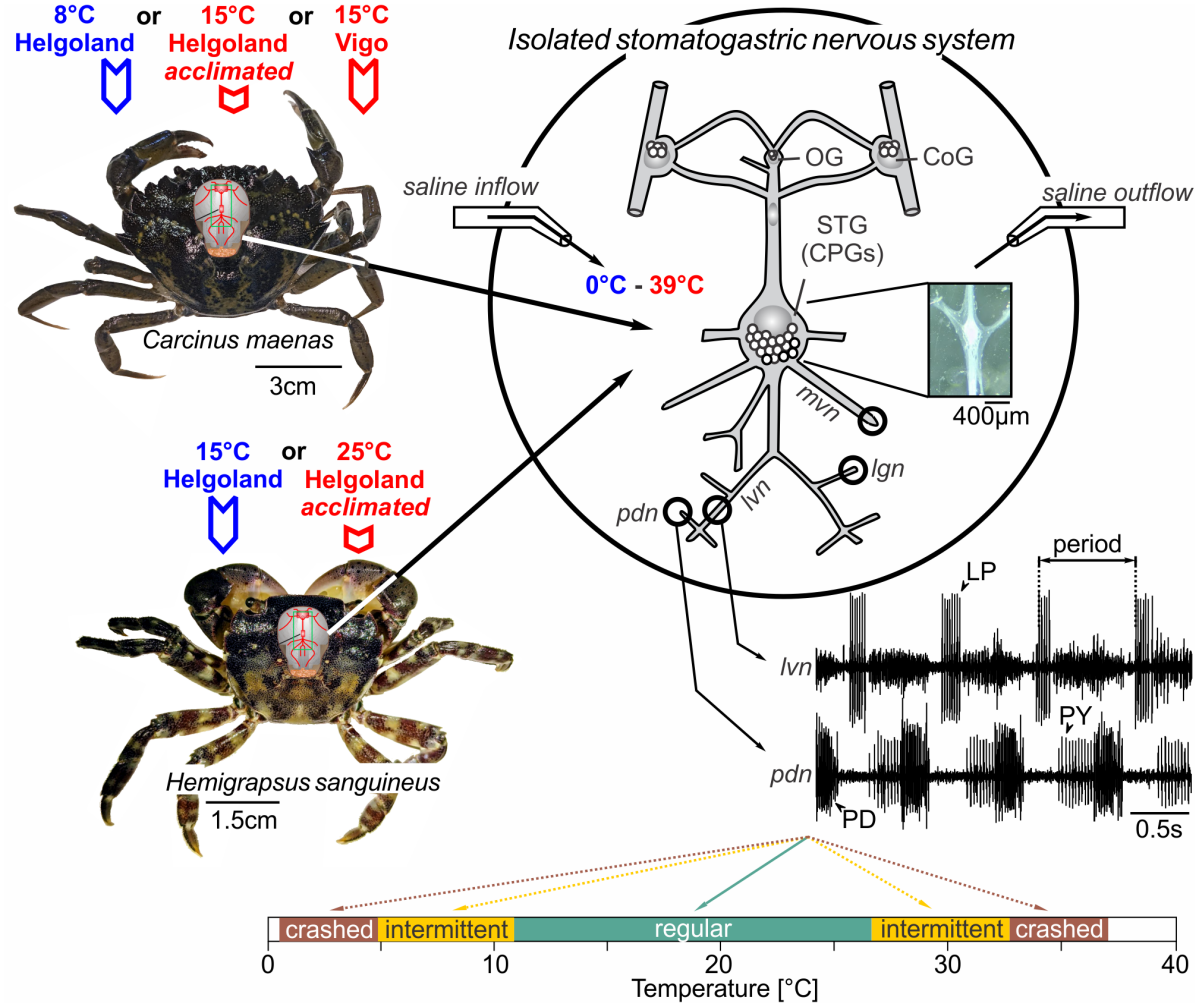
The stomatogastric nervous systems of adult *H. sanguineus* and *C. maenas* crabs were dissected and placed in a saline-superfused Petri dish. The isolated nervous system was continuously superfused with temperature-controlled saline (ramps between 0°C and 39°C). During the experiment, extracellular recordings of the *lvn* and *pdn* were carried out to access the activities of the pyloric LP, PY and PD neurons. In some experiments, the *mvn* and *lgn* were also recorded to monitor gastric mill motor neurons. Extracellular recordings were achieved by placing petroleum jelly wells around the respective nerves. Bottom: rhythms were classified into the following categories: ruby, absent rhythmicity (‘crash’); orange, intermittent activity with one neuron type failing to fire or with intermittent rhythmicity; teal: “regular” with rhythmic activity and constant phase relationships. The outer boundaries of the ruby zones mark the minimum and maximum temperatures tested. OG, oesophageal ganglion; CoG, commissural ganglion; STG, stomatogastric ganglion; LP, lateral pyloric neuron; PY, pyloric constrictor neurons; PD, pyloric dilator neurons; *lvn*, lateral ventricular nerve; *pdn*, pyloric dilator nerve; *lgn*, lateral gastric nerve; *mvn*, medial ventricular nerve.

Extracellular recordings were performed from nerves via stainless steel pin electrodes (Städele et al., 2017). Stretches of the lateral ventricular nerve (*lvn*), pyloric dilator nerve (*pdn*), pyloric constrictor nerve (*pyn*), medial ventricular nerve (*mvn*), and lateral gastric nerve (*lgn*) were electrically isolated from the bath with petroleum jelly wells (Figure 1). Action potentials were recorded, amplified, and filtered using Isleworth electronics amplifiers and digitized at 22 kHz through a Behringer U-Control UCA222 USB Audio Interface. Example recordings of the *pdn* and *lvn* are given in Figure 1.

### Data analysis, statistical analysis

Electrophysiological files were recorded and saved using WinEDR (version 4.0, Strathclyde Electrophysiology Software). Files were analyzed in Spike 2 (version 7.18, CED) using original Spike 2 scripts, after conversion to Spike 2 format. All neurons could be individually identified on the respective nerve recordings. Cycle periods and cycle frequencies (1/cycle period) were calculated from the beginning of one burst of the pyloric dilator neuron (PD) to the beginning of the next burst. Duty cycles and phase relationships were calculated by dividing burst parameters (duration, start, end) by cycle period.

We used parametric tests (ANOVA and t-test) for normally distributed data. For non-normally distributed data, we used Friedman Analysis of Variance on Ranks. In cases where multiple variables were measured in the same animals, repeated measures tests were considered. The factors and specific designs of each test are given in Supplementary Data Sheet 1. Statistical tests were performed using SigmaStat (version 11; Systat Software, San Jose, CA). For ANOVA, statistical tests are reported in the format: statistical test, F (degrees of freedom, residual) = *F* value, *p* value, *post hoc* test. “N” denotes the number of preparations. *Post hoc* tests are at a significance level of 0.05. Data was prepared in Excel and finalized in Coreldraw (version X7; Corel Inc.). In figures, data is presented as mean ± SD or mean ± SEM unless otherwise noted.

### Experiments and definitions

We define robustness as the tolerance of the pyloric network against acute temperature perturbations. Specifically, we classified pyloric rhythms as “regular” when they were triphasic and continuously active, i.e., when the three main pyloric neurons showed continuous regular and rhythmic burst activity (see Figure 1, bottom for example). We deemed rhythms “intermediate” when activity patterns were rhythmic, but failed to show all three phases, i.e., when one or more main pyloric neurons no longer produced regular bursts. Finally, rhythms were considered to have “crashed” when they became arrhythmic. Intermittent and crashed conditions could occur through the loss of action potentials, or the inconsistent bursting of pyloric neurons, among others. To compare between the different categories, rhythms were also color-coded (Figure 1, bottom). We compared temperature robustness in several conditions. In experiment 1, two different temperature ramps were applied: Starting from 15 to 20°C, the temperature was lowered first (“cold ramp”) and heated after (“hot ramp”). For cold ramps, the temperature was lowered just beyond the cold crash point at which rhythmic pyloric activity stopped. Because the temperature in the experimental room varied, the cold crash temperature could not be reached in all experiments, although the cooling system operated at maximum capacity. For hot ramps, the temperature was increased just beyond the temperature at which the hot crash occurred, and rhythmic pyloric activity seized. Care was taken not to heat the preparation above 39°C to not damage the neural tissue. In experiment 2, ramp order was reversed, and the preparation was heated first and cooled after. In experiment 3, animals were warm-acclimated in the laboratory for at least 2 months before dissection and being tested with cold and hot ramps (in that order). In experiment 4, we exposed animals from Spain and acclimatized to warmer temperatures to cold and hot ramps.

### Temperature control

Preparations were continuously superfused with physiological saline (7-12 mL/min, Figure 1). Saline superfusion was temperature controlled by a Peltier device (AC Alseye Wasserkühlung Halo H360). Temperature was altered between 0 and 39°C and changed by approximately 1–2°C/min, mirroring previous studies (Städele et al., 2015). Temperature was continuously measured close to the STG with a temperature data logger (Dostmann LOG200 TC, one sample per second). Saline inflow to the nervous system was positioned within 5–7 mm of the STG so that the measured temperature at the inflow point was approximately that of the ganglion.

## Results

To identify the range in which *H. sanguineus* and *C. maenas* pyloric activity remained regular, we recorded the three main pyloric neuron types – the pyloric dilator neurons (PD), the single lateral pyloric neuron (LP) and the pyloric constrictor neurons (PY) – and recorded their spontaneous activities at different temperatures (Figure 1). These three neuron types have been identified in all previous studies of the decapod stomatogastric nervous system (Stein, 2017). Together, they produce the pyloric motor pattern that drives the filtering of food in the pylorus, separating large food particles in the stomach from smaller ones that move on to the midgut. Previous studies have demonstrated that the pyloric motor pattern is triphasic with fixed phase relationships. The phase relationships are recognizable in all animals of a given species (Bucher et al., 2005), and studies on the Jonah crab, *Cancer borealis*, and the brown crab, *Cancer pagurus*, have shown they are also maintained over a wide temperature range (Tang et al., 2010; Soofi et al., 2014).

### Characterization of stomatogastric neuronal activity in *Hemigrapsus sanguineus* and *Carcinus maenas*

**Figure 2:**
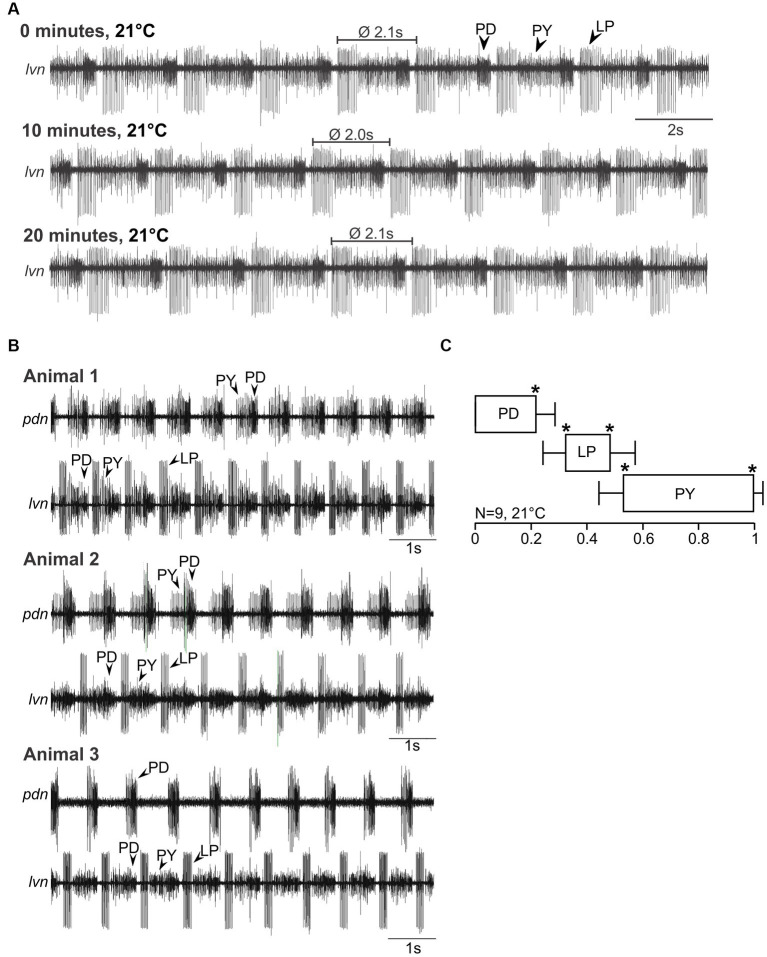
Spontaneous pyloric activity in adult *H. sanguineus* is stable and consistent over time and between animals. **(A)** Spontaneous pyloric rhythm at 21°C, recorded on the *lvn*, with regular LP, PY, and PD activities. Recordings were taken at time 0, then 10 min later, and at 20 min. **(B)** Comparison of spontaneous pyloric rhythms from three animals, recorded at 21°C from the *pdn* and the *lvn*. All animals showed regular and long-lasting rhythmic pyloric activity. **(C)** Phase diagram of the pyloric neurons, averaged from *N* = 9 animals with spontaneous pyloric rhythm, at 21°C. *p < 0.05. Friedman Repeated Measures Analysis of Variance on Ranks, Chi-square = 45.000, df = 5, *p* < 0.001. All phase values were significantly different from each other (SNK post-hoc test, *p* < 0.05).

Since no previous study has recorded pyloric activity in *H. sanguineus* and *C. maenas*, we first characterized the activity of the pyloric neurons and determined if it was generated consistently and reliably in both species. Figure 2A shows a recording in an isolated *H. sanguineus* nervous system (Figure 1), at 21°C at 0, 10 and 20 min. The pyloric rhythm was immediately recognizable and stable across all time points. Like in other species, PD led the cycle, followed by LP and PY (Figure 2A, top trace). This triphasic pattern repeated itself every 2.1 s, i.e., at the pyloric cycle period. The pyloric rhythm continued in a stereotypical fashion as long as no experimental manipulation was applied (Figure 2A, middle and bottom traces).

The pyloric rhythm in Figure 2A is representative of the typical spontaneous activity found in all animals of that species. Figure 2B shows recordings from three different animals. Here, we additionally recorded a second, more posterior nerve to allow a better separation of the activities of the individual neuron types, and to create a phase plot. We named this nerve the pyloric dilator nerve (*pdn*) based on its anatomical features, the muscles it innervates, and its similarity with previously studied *pdn* of other species (Maynard and Dando, 1974). However, while in other species the *pdn* exclusively contains the PD neurons, we also picked up PY activity in about half of our *pdn* recordings. This might have been due to the smallness of the nerve and crosstalk from the pyloric constrictor nerve (*pyn*), an incomplete separation of the *pyn* from the *pdn*, or because in this species, no clearly separatable *pyn* exists (i.e., *pyn* and *pdn* may be conjoint). Nevertheless, the different action potential amplitudes and firing patterns allowed a straight-forward separation of the three pyloric neuron types. As evident from the recordings of the three animals in Figure 2B, the pyloric pattern was consistent and recognizable across multiple individuals of *H. sanguineus.* Accordingly, the phase plot (Figure 2C) shows three clearly separated activity phases (PD, LP and PY). Like in other decapod crustaceans, neuronal activity phases started and ended at significantly different phase values, with low variability between individuals.

**Figure 3:**
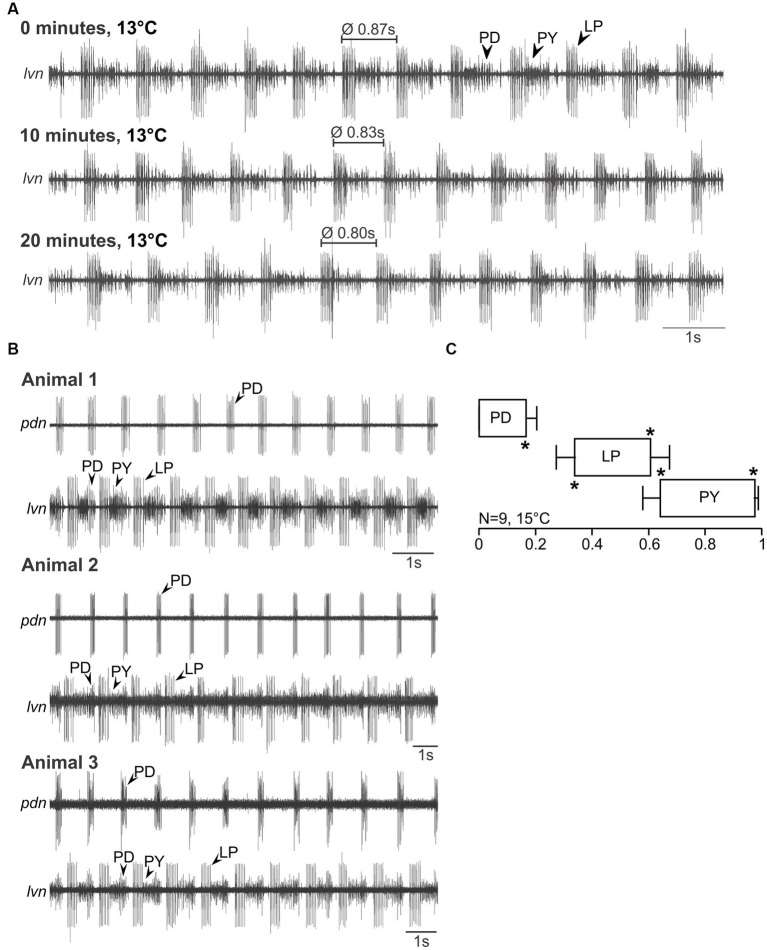
Spontaneous pyloric activity in adult *C. maenas* is stable and consistent over time and between animals. **(A)** Spontaneous pyloric rhythm at 13°C, recorded on the *lvn*, with regular LP, PY, and PD activities. Recordings were taken at time 0, then 10 min later, and at 20 min. **(B)** Comparison of spontaneous pyloric rhythms from three animals, recorded at 15°C from the *pdn* and the *lvn*. All animals showed regular and long-lasting rhythmic pyloric activity. **(C)** Phase diagram of the pyloric neurons, averaged from *N* = 9 animals with spontaneous pyloric rhythm, at 15°C. *p < 0.05. Friedman Repeated Measures Analysis of Variance on Ranks, Chi-square = 45.000, df = 5, *p* < 0.001. All phase values were significantly different from each other (SNK post-hoc test, *p* < 0.05).

For *C. maenas*, we found qualitatively similar results. Figure 3A shows a recording of the *lvn* in an isolated nervous system at 0, 10, and 20 min (13°C). The pyloric rhythm was recognizable, showed the familiar PD, LP, and PY phases, and was stable over time (Figure 3A, middle and bottom traces). Figure 3B shows the spontaneous pyloric activity of three animals (*C. maenas*), recorded on the *lvn* and *pdn*, demonstrating that all *C. maenas* generate the canonical pyloric rhythms observed in other decapod crustaceans. Unlike in *H. sanguineus*, however, the *pdn* never contained PY activity, suggesting that there is a complete separation between *pdn* and *pyn* in this species. In fact, in a few preparations, we recorded the *pyn* instead or in addition to the *pdn*, and it contained only the PY neuron activity, but never the PDs. The phase plot (Figure 3C) shows three distinct activity phases (PD, LP, and PY), with significantly different phase values and low variability.

Besides the pyloric rhythm, we also detected the presence of the slow gastric mill rhythm in both species. The gastric mill rhythm is episodic and depends on extrinsic neuromodulatory input from descending projection neurons in the commissural ganglia (Nusbaum and Beenhakker, 2002; Stein, 2009; Stein, 2017; Blitz, 2023). In most studied species, it is thus not continuously active in isolated nervous system preparations but can occur spontaneously. This was also the case in *H. sanguineus* and *C. maenas*. In a subset of animals, we found spontaneous gastric mill rhythms (Supplementary Data Sheet 2), at various temperatures (typically during cold ramps). The temperature robustness of the gastric mill rhythm is under active investigation (Städele et al., 2015; DeMaegd and Stein, 2021; Powell et al., 2021; Stadele and Stein, 2022), and depends on extrinsic modulatory input that is needed to enable this rhythm.

### Neuronal responses to acute temperature challenges in *Hemigrapsus sanguineus*

**Figure 4:**
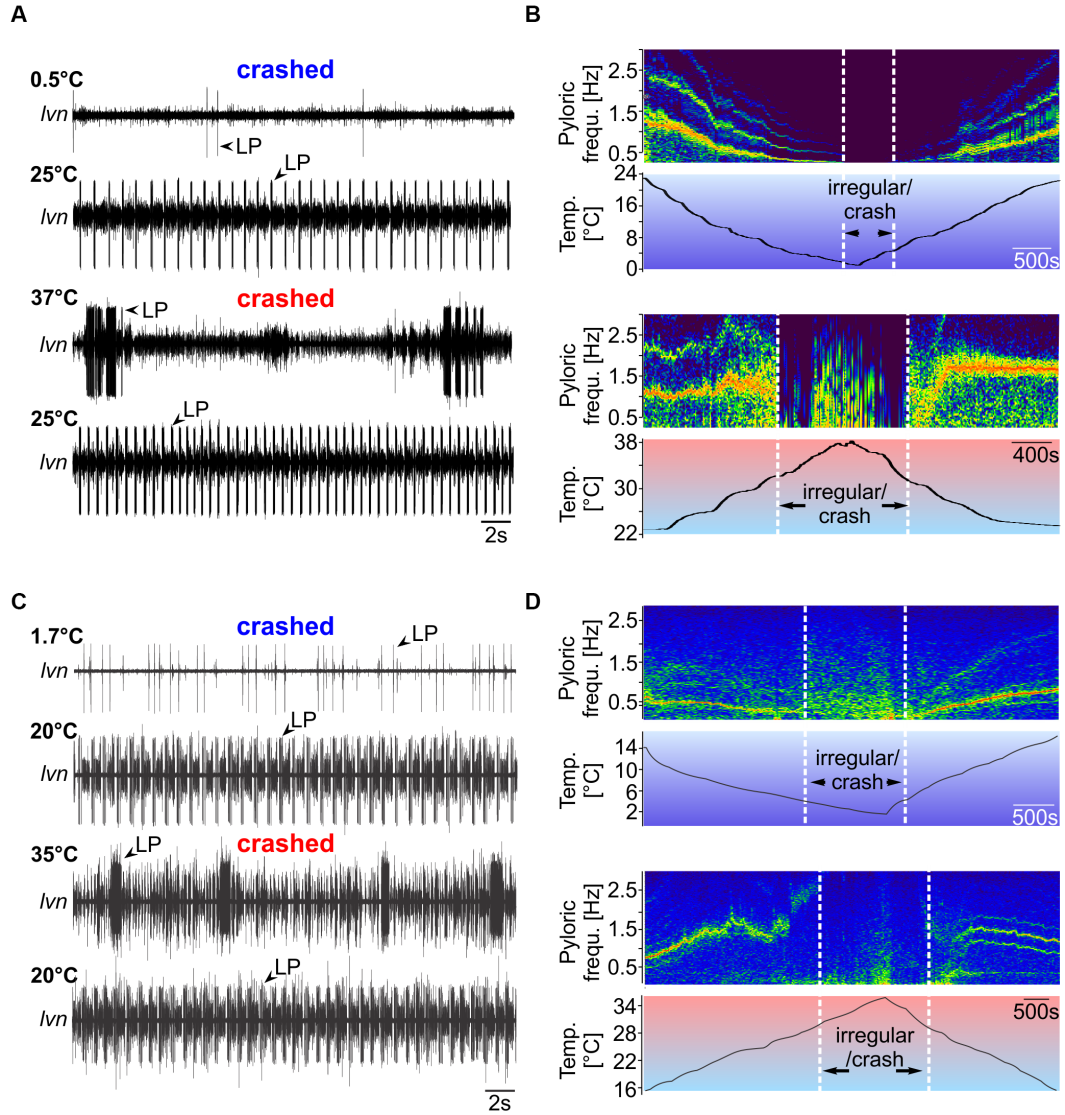
Spontaneous pyloric rhythms in *H. sanguineus* and *C. maenas* crash at temperatures close to 0°C and 40°C, respectively, but recover from cold and heat treatments. **(A)** Pyloric activity at 0.5°C, 25°C, 37°C, and 25°C in *H. sanguineus*. The rhythm was absent (‘cold crash’) at 0.5°C, and at 37°C (‘heat crash’). Crashes were reversible and caused no permanent damage. At 25°C, a regular rhythm had returned. This was the case after cold and hot treatment. **(B)** Spectrograms showing the main frequency components of the pyloric rhythm over temperature. Hotter colors indicate more spectral power. When cooled down (top spectrogram), the rhythm slowed down continuously until individual neuron types failed to fire regularly and rhythmicity stopped (‘irregular/crash’). The crash during the hot ramp (bottom spectrogram) occurred through a sudden loss of coordinated activity, followed by irregular firing of various neuron types. Plots were cut off above the fundamental frequency bands to avoid plotting harmonics for clarity. **(C)** Pyloric activity at 1.7°C, 20°C, 35°C, and 20°C in *C. maenas*. The rhythm was absent (‘cold crash’) at 1.7°C, and at 35°C. Crashes were reversible and caused no permanent damage. At 20°C, a regular rhythm had returned. This was the case after cold and hot treatment. **(D)** When cooled down (top spectrogram), the rhythm slowed down continuously until individual neuron types fired irregularly. At this point, rhythmicity was absent (‘irregular/crash’). During the hot ramp (bottom spectrogram), pyloric cycle frequency increased until a sudden loss of coordinated activity occurred. After the hot crash, rhythmicity returned.

In experiment 1, we tested temperature robustness by measuring the temperature range in which the pyloric rhythm remained congruent. For this, we applied temperature ramps starting from the animals’ habitat temperature. Ramps were introduced by cooling or heating the inflowing saline (see Material and Methods). Figure 4A shows the result of a temperature ramp experiment in *H. sanguineus*. We first cooled the preparation from room temperature (21°C) to 0°C, then heated it to 39°C, and then cooled it back down to 21°C. Three temperatures are shown (0.5°C, 25°C and 37°C, and the post-heat condition of 25°C). At temperatures close to 0°C, the rhythm became slow, and eventually disorganized. Individual neuron types failed to produce action potentials, with LP typically being the first neuron to be missing, while PD and PY continued to generate alternating rhythmic activity, albeit at a very slow rate. Eventually, rhythmicity seized entirely (Figure 4A, top recording). Cold treatment did not permanently damage the rhythm and we did not cool enough to cause icing. When heated back up, the rhythm returned, and all neuron types recovered. An example from 25°C after cold treatment is shown in Figure 4A (second recording from the top), showing strong and regular rhythmic activity of all pyloric neuron types. When heated up further, the rhythm became irregular, with individual neuron types failing or being tonically active. The behavior of the pyloric neurons at high temperatures varied substantially between animals, as previously reported for other species (Tang et al., 2012; Marder and Rue, 2021), ranging from irregular bursting with intermittent tonic activity (Figure 4A, third recording) to tonic activity and complete absence of multiple neuron types (examples are given in Supplementary Data Sheet 3). The heat-induced breakdown of rhythmic pyloric activity was also not permanent, and when cooled back down, rhythmicity was restored (Figure 4A, bottom trace). There was an apparent hysteresis of the pyloric cycle frequency, in that the pyloric rhythm was faster after it recovered from the heat-induced crash. Figure 4B shows spectrograms of the cold (top) and hot (bottom) ramps of one experiment, with warmer colors indicating more spectral power and highlighting the main frequency components of the pyloric rhythm. During the cold ramp, pyloric cycle frequency decreased steadily, until rhythmicity ended (left vertical white bar). Rhythmicity resumed (right vertical bar) and cycle frequency increased steadily when the temperature was raised again. During the hot ramp, pyloric cycle frequency remained remarkably stable, and decreased slightly just before rhythmicity ended (left vertical bar). Further heating the preparation resulted in activities with frequency gaps or multiple frequency bands, i.e., variable spiking with intermittent bursting of the pyloric neurons, indicating that the rhythm had crashed. When cooled back down, rhythmic activity resumed (right vertical bar) with moderately varying, slow cycle frequencies. Eventually, the rhythm settled into consistent rhythmicity with a stable cycle frequency nearly independent of temperature.

### Neuronal responses to acute temperature challenges in *Carcinus maenas*

We found similar pyloric responses for *C. maenas* (Figure 4C). When cooled to 1.7°C, the rhythm was absent (top trace), but recovered when heated again (second trace). When heated to 35°C, the rhythm failed and individual neurons fired at varying rates with no apparent rhythmicity. LP, for example, fired in strong bursts with variable interburst intervals. Like in *H. sanguineus*, the behavior of the pyloric neurons at high temperatures varied dramatically between animals. Crash activity ranged from irregular bursting with intermittent tonic activity (Figure 4C, third recording) to tonic activity and complete absence of multiple neuron types (Supplementary Data Sheet 4). The spectrograms (Figure 4D) demonstrate that during the cold ramp, cycle frequency decreased steadily until rhythmicity seized. When warmed up after the cold crash, cycle frequency increased again steadily. During the hot ramp, cycle frequency continued to increase (in contrast to *H. sanguineus*) to roughly 2.5 Hz before rhythmicity stopped and the rhythm crashed. The rhythm resumed after cooling the preparation.

### Changes to pyloric phase relationships during acute temperature challenges in *Hemigrapsus sanguineus*

**Figure 5:**
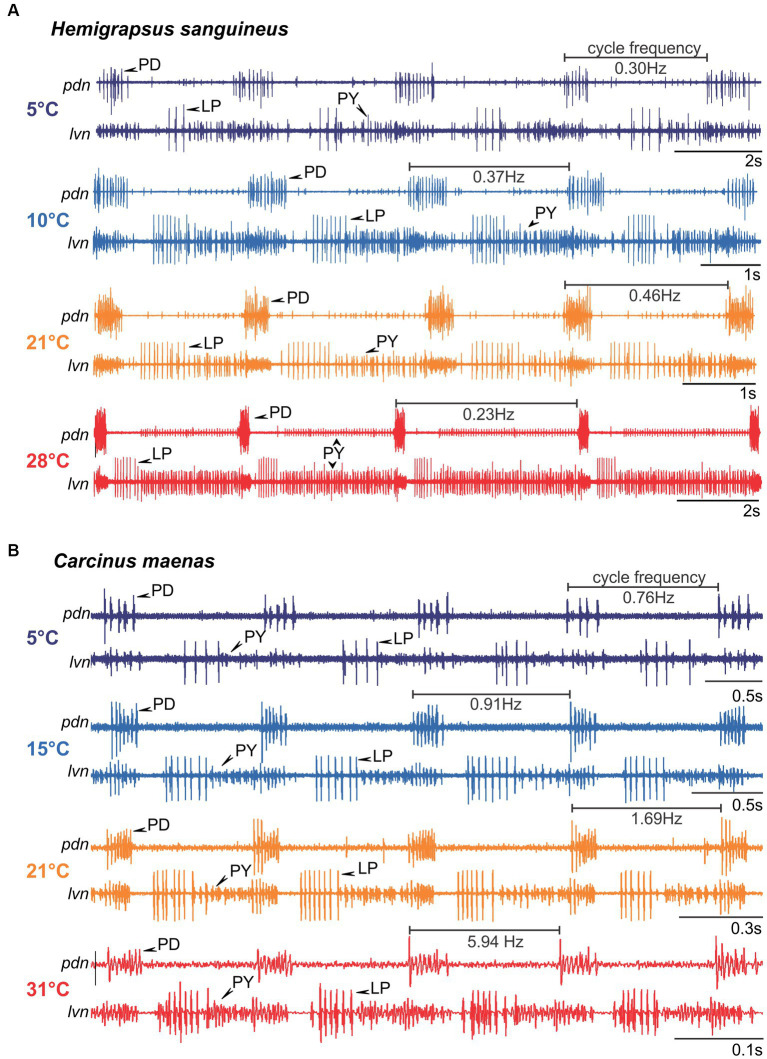
Original recordings of the pyloric rhythm in *H. sanguineus* **(A)** and *C. maenas* **(B)** at different temperatures. The cycle frequency of the rhythm at each temperature is given. Note the difference in the time scale bars. For clarity, each recording was scaled so that 4 pyloric cycles are shown and the phase relationship between pyloric neurons are recognizable. Rhythms were regular at all shown temperatures, but phase relationships changed.

**Figure 6:**
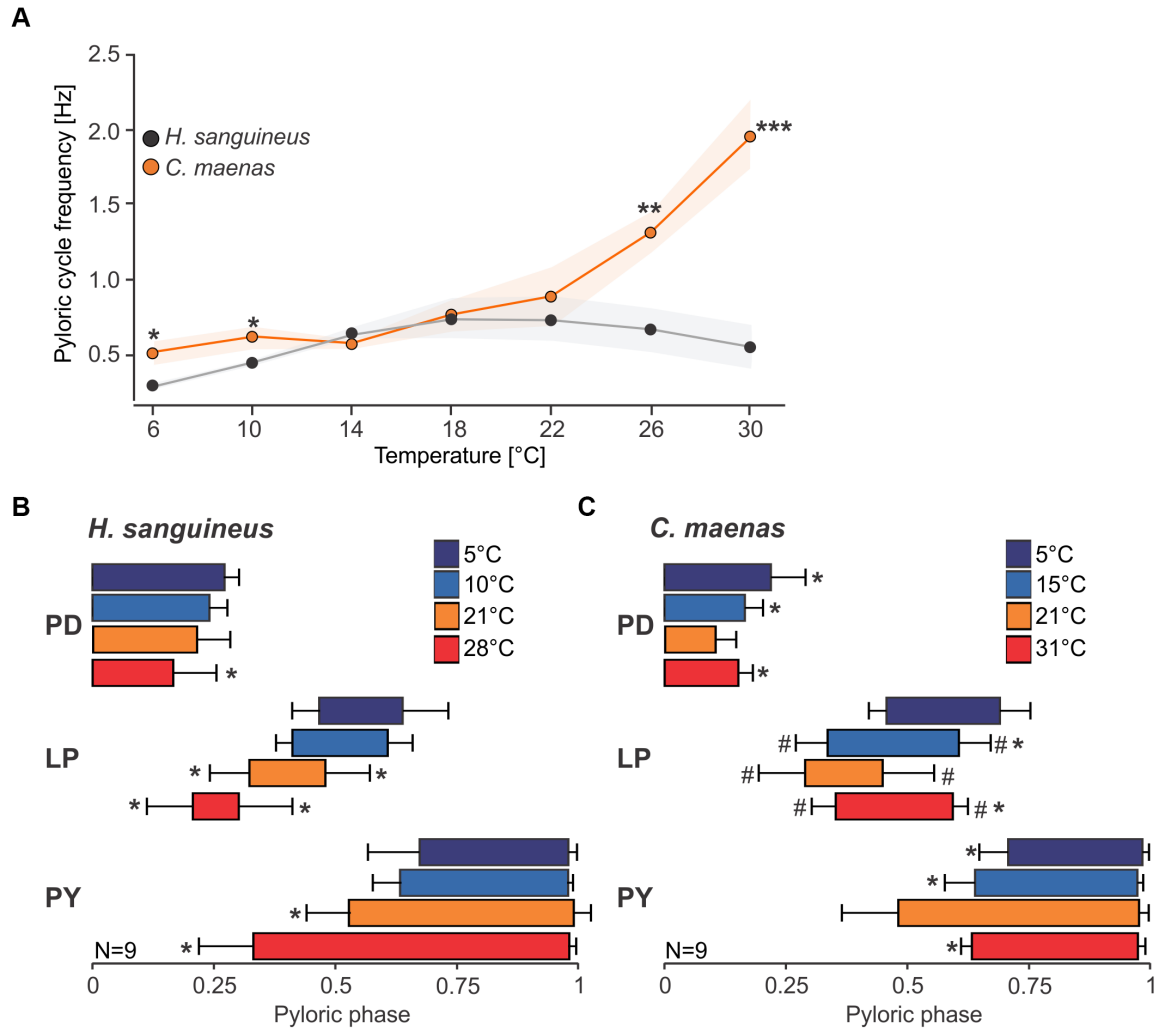
Pyloric cycle frequency and phase relationships change with temperature. **(A)** Analysis of pyloric cycle frequency in *H. sanguineus* (gray) and *C. maenas* (orange). In *C. maenas*, cycle frequency was remarkably stable at cold temperatures, but increased dramatically at warm temperatures. In contrast, cycle frequency in *H. sanguineus* diminished at cold and warm temperatures, leading to significant differences between the two species. *p < 0.05, **p < 0.01, ***p < 0.01, *t*-test, Supplementary Data Sheet 1I. Means±SEM are shown. **(B)** Phase plot of pyloric neurons in *H. sanguineus*, at different temperatures. Temperatures are color-coded. *p < 0.05, significantly different from 5°C and 10°C, Supplementary Data Sheets 1II–VI. **(C)** Phase plot of pyloric neurons in *C. maenas*, at different temperatures. Temperatures are color-coded. *p < 0.05, significantly different from 21°C. #*p* < 0.05, significantly different from 5°C, Supplementary Data Sheets 1VII–XI. Means±SD are shown.

The spectrograms indicated that temperature responses of the cycle frequency and the phase relationships between pyloric neurons might be strikingly different between species. We thus investigated the pyloric cycle frequency and phase relationships in more detail. Figure 5 shows original recordings of the pyloric rhythm in *H. sanguineus* (Figure 5A) and *C. maenas* (Figure 5B), at various temperatures at which the rhythm was continuous and regular. In *H. sanguineus*, the pyloric cycle frequency was slow at 5°C, then increased at 10°C and 21°C, but surprisingly slowed down again at 28°C. This was consistent across animals (Figure 6A) and unlike all other tested species before, where cycle frequency continues to increase until the heat crash occurs (Tang et al., 2010; Soofi et al., 2014). Likewise, the phase relationships between the pyloric neurons changed significantly with temperature, unlike previously studied species where phase relationships remained constant over a broad temperature range (Tang et al., 2010, Soofi et al., 2014). Specifically, rising temperatures shortened the PD duty cycle, such that the PD bursts terminated earlier during the cycle. This can be seen in the shortened PD bursts at 28°C within the pyloric cycle (Figure 5A) and in a significantly earlier end of the PD burst in the phase diagram (Figure 6B). The LP duty cycle also shortened at higher temperatures, and the LP burst started and ended significantly earlier during the cycle (Figure 6B). The most dramatic change was in the duty cycle of the PY neurons. Their relative contribution to the pyloric cycle increased with higher temperatures, as can be seen in the much longer PY bursts at 21°C and 28°C (Figure 5A). The phase diagram shows that the increased duty cycle was entirely due to an earlier start of the PY burst during the cycle (Figure 6B).

### Changes to pyloric phase relationships during acute temperature challenges in *Carcinus maenas*

In *C. maenas*, cycle frequencies changed only modestly between 5°C and 15°C, but then increased dramatically with higher temperatures. In the example shown in Figure 5B, cycle frequency reached almost 6 Hz (= 6 cycles per second) at 31°C, compared to 0.76 Hz (= less than one cycle per second) at 5°C. The quantitative analysis of the cycle frequency corroborated these observations (Figure 6A). The modest decrease in cycle frequency at cold temperatures and strong increase at warm temperatures contrasted with *H. sanguineus*: While there was no significant difference in cycle frequency between the two species in the medium temperature range (14°C - 22°C), cycle frequencies in *C. maenas* were significantly higher at cold and warm temperatures (Figure 6A). We also detected temperature-dependent changes to the pyloric phase relationships in *C. maenas*, albeit different from those in *H. sanguineus*. The main change in the phase relationships occurred in the medium temperature range, particularly at 21°C. Here, the duty cycle of the PD burst was the shortest relative to the cycle period, and the LP burst occurred earlier than at other temperatures. Accordingly, the PY burst occurred earlier as well, and its duty cycle was prolonged. This can be seen in the relatively long PY bursts in Figure 5B at 21°C, and the significantly earlier PY onset in the phase diagram (Figure 6C). The phase diagram also shows that temperature changes affected LP the most. At 15°C, 21°C, and 31°C, the start and end of its activity phase occurred significantly earlier than at 5°C. At 21°C, the end of its activity phase was significantly earlier than at 15°C and 31°C (Figure 6C). The latter corresponded well with the significantly earlier start of the PY activity phase at 21°C (in comparison to all other temperatures).

### Robustness to acute temperature challenges in *Hemigrapsus sanguineus*

Overall, both species showed a regular pyloric rhythm over a wide range of temperatures (around 30°C), even though phase relationships and cycle frequency changed and how they changed was distinct between species. The most dramatic change to pyloric activity occurred when the rhythm stopped at the low and high ends of our temperature ramps. To further analyze the temperature robustness of the pyloric rhythm, we thus classified pyloric activity (“regular,” “intermittent,” and “crashed”; see Methods and Figure 1, bottom) and color-coded it. The data shown was collected by exposing the STNS to cold ramps first, followed by hot ramps (experiment 1). We tested whether the order of the ramps (cold first, hot second or vice versa) affected the hot and cold crash points by carrying out additional, reversed order ramps in a small set of experiments (experiment 2, Supplementary Data Sheet 5). This was not the case.

**Figure 7:**
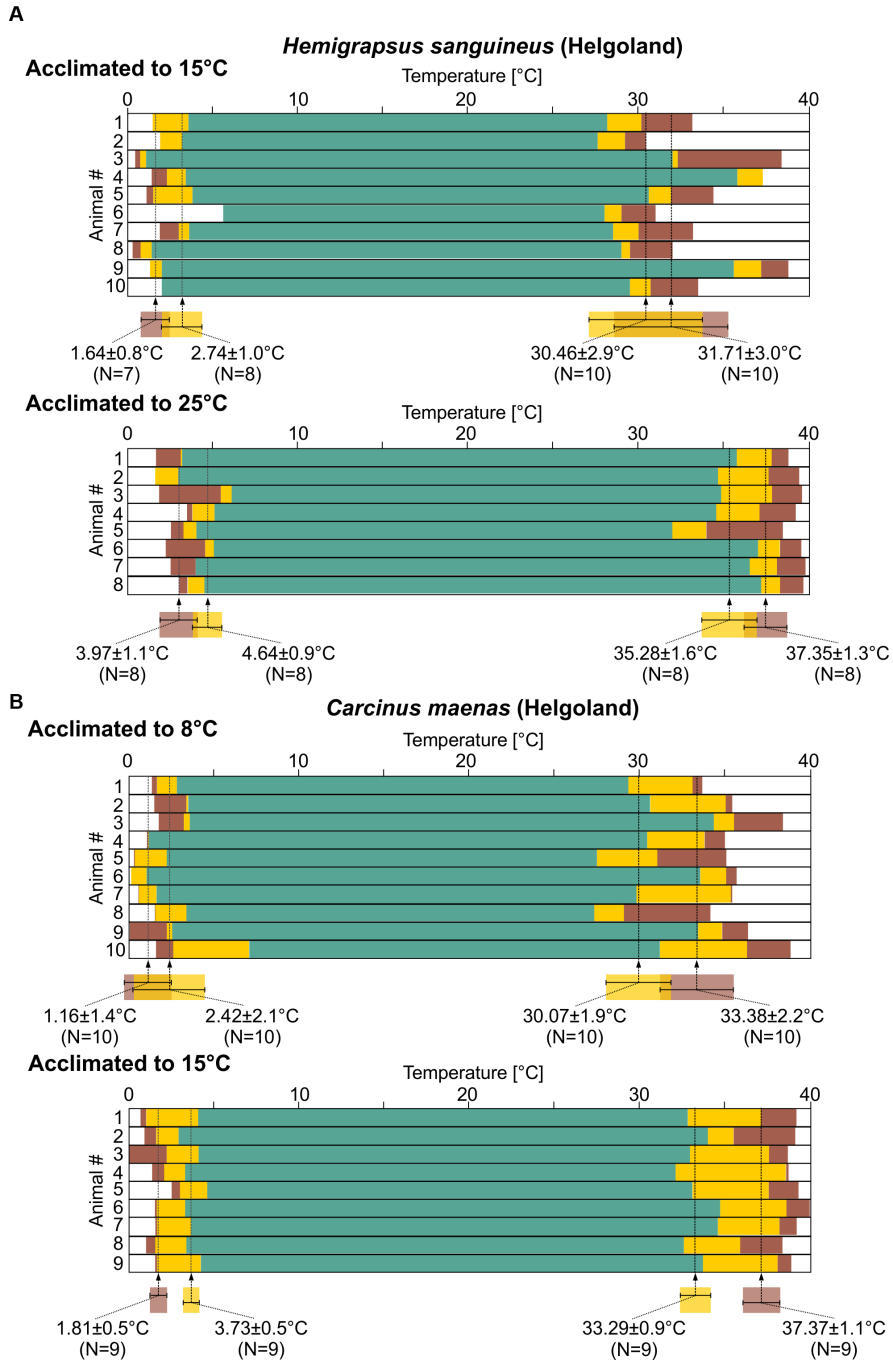
The pyloric rhythms in *H. sanguineus* and *C. maenas* show a broad temperature robustness. Summary diagrams of pyloric activity over temperature (see Figure 1, bottom). Statistical significances are given in Supplementary Data Sheets 1XII–XV. **(A)** Top: 10 *H. sanguineus* recordings from animals caught in Helgoland and acclimated to 15°C. Means±SD of temperatures at which the activity transitioned into a different state are given along with N numbers that could be analyzed. Bottom: 8 *H. sanguineus* recordings (Helgoland, acclimated to 25°C). **(B)** Top: 10 *C. maenas* recordings (Helgoland, acclimated to 8°C). Bottom: 9 *C. maenas* recordings (Helgoland, acclimated to 15°C).

Figure 7A shows the summary data for 10 experiments with *H. sanguineus*. These animals had been collected in Helgoland, where the average water temperature was 15°C. After being collected, they had been kept at 15°C in single tanks for at least 2 months, until they were used in the experiment. In all tested animals, the pyloric rhythm showed regular activity over a wide range of temperatures, i.e., the rhythm had a substantial temperature robustness. On average, the pyloric rhythm left the teal, regular condition at 2.74 ± 1.0°C (*N* = 8) during cold ramps, and finally failed entirely (crashed) at 1.64 ± 0.8°C (*N* = 7). Irregular or crashed conditions could not be achieved in all preparations: In two preparations we could not cool the preparation enough to reach intermittent activity, and in one additional preparation we did not reach the cold crash point. These data were thus excluded from the calculation of the average temperatures. During hot ramps, the pyloric rhythm remained regular up to 30.46 ± 2.9°C (*N* = 10), after which it entered the intermittent activity condition. It crashed at 31.71 ± 3.0°C (*N* = 10). There was substantial variability of the crash temperatures between individual animals, much more so than during cold ramps, as indicated by the approximately three-fold larger standard deviation during the hot ramp.

### Temperature acclimation of neuronal responses in *Hemigrapsus sanguineus*

In its native range, *H. sanguineus* experiences temperatures between 0 and 23°C throughout the year (Fukui, 1988; Stephenson et al., 2009). In experiment 3, we searched for a potential acclimation in *H. sanguineus* by warm-acclimating the batch of *H. sanguineus* that were collected at 15°C to 25°C for at least 2 months. On average, the pyloric rhythm left the teal, regular condition at 4.64 ± 0.9°C (Figure 7A; *N* = 8) during cold ramps and crashed at 3.97 ± 1.1°C (*N* = 8). Both temperatures were significantly higher than in the 15°C group (Supplementary Data Sheets 1 XII, XIII). This is consistent with our observation that we were able to reach the cold crash point in all 8 tested animals (as opposed to the 15°C group), because the preparations required less cooling. During hot ramps, the pyloric rhythm remained regular up to 35.28 ± 1.6°C (*N* = 8), after which it entered the intermittent activity condition. It crashed at 37.35 ± 1.3°C (*N* = 8). There was variability between individual animals: For cold ramps, inter-animal variability appeared similar to the 15°C group, but variability was reduced for hot crashes compared to the 15°C group. In fact, in the 25°C group, the variability for the hot crashes now seemed similar to that for the cold crashes. In summary for *H. sanguineus*, all crash temperatures were significantly shifted after warm acclimation (Supplementary Data Sheets 1XII–XV).

### Robustness to acute temperature challenges in *Carcinus maenas*

For *C. maenas*, experiment 1 used animals that were collected in Helgoland, at an average water temperature of 8°C, and were then kept at 8°C for at least 2 months. During cold ramps, the pyloric rhythm remained in the teal, regular condition until the temperature reached 2.42 ± 2.1°C (Figure 7B, top; *N* = 10). It crashed at 1.16 ± 1.4°C (*N* = 10). During hot ramps, it entered the intermittent activity condition at 30.07 ± 1.9°C (*N* = 10), and crashed at 33.38 ± 2.2°C (*N* = 10). There was substantial variability in crash temperatures between individual animals, during both cold and hot ramps.

### Temperature acclimation of neuronal responses in *Carcinus maenas*

We then acclimated animals for experiment 3 to 15°C for at least 2 months before exposing them to cold and hot ramps (Figure 7B, bottom). During cold ramps, the rhythm remained regular until a temperature of 3.73 ± 0.5°C (*N* = 9) was reached. It crashed at 1.81 ± 0.5°C (*N* = 9). During hot ramps, the rhythm remained in the teal, regular condition up to 33.29 ± 0.9°C (*N* = 9). The intermittent activity condition was observed up to 37.37 ± 1.1°C (*N* = 9) above which the rhythm crashed. The variability of the crash temperatures between individual animals was lower than in cold-acclimated animals, with the standard deviation being about half of that of the cold-acclimated group. The hot crash temperature thus shifted significantly after acclimation (Supplementary Data Sheets 1XII–XV). For cold ramps, the temperature at which the intermittent activity started was significantly higher, but the ultimate cold crash temperature remained unaffected.

### Comparison of temperature robustness between populations of *Carcinus maenas*

**Figure 8:**
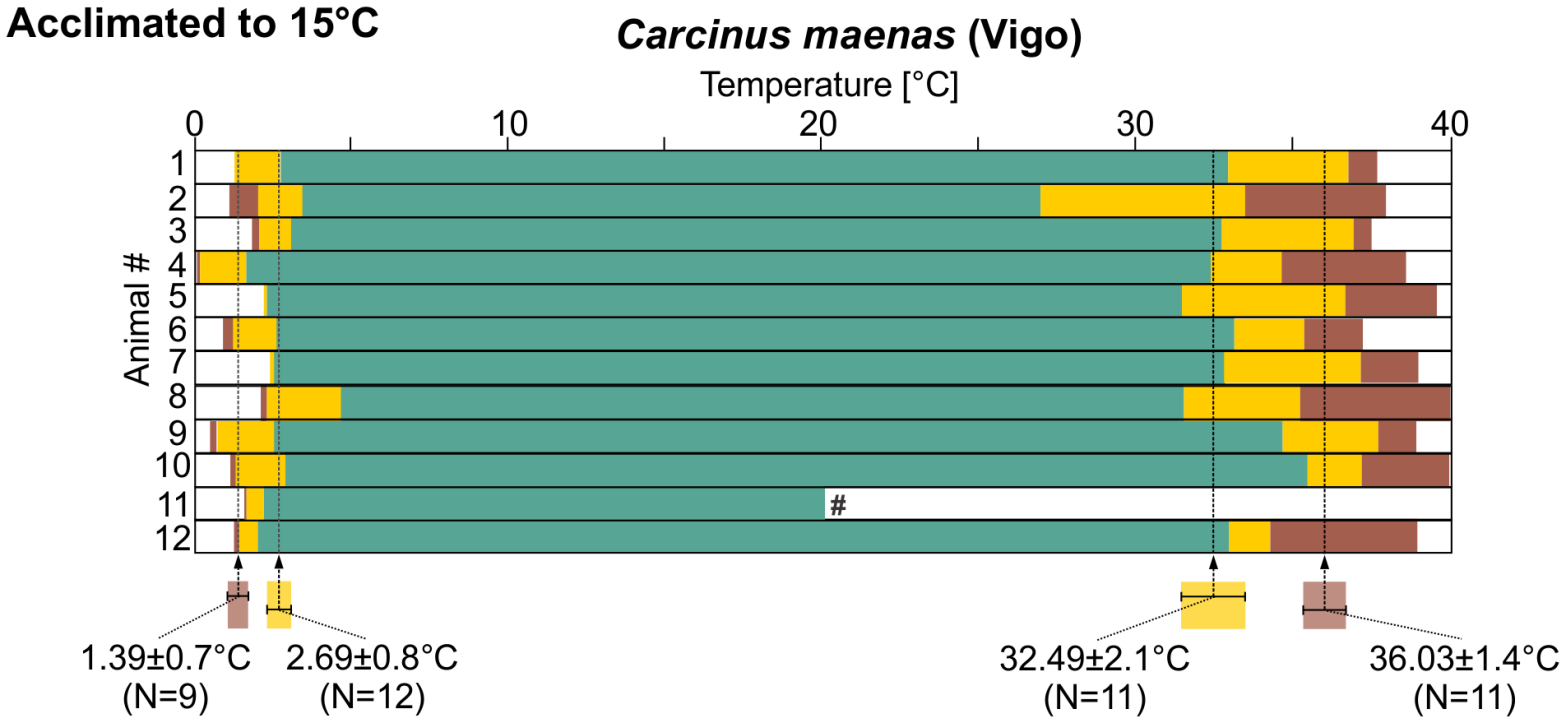
Summary diagrams of pyloric activity over temperature for 12 *C. maenas* recordings from animals that were collected in Vigo and acclimated to 15°C. Means±SD of temperatures at which the activity transitioned into a different state are given along with the N numbers that could be analyzed. Statistical significances are given in Supplementary Data Sheets 1XII–XV. #, this recording was lost after the cold ramp.

In the experiments so far, we had collected one batch of animals and then acclimated them to a new, higher habitat temperature in the lab. We predicted that animals in the wild would display similar acclimatization capabilities. To test this prediction, in experiment 4 we collected *C. maenas* from Vigo (Spain), where the average habitat temperature was 15°C, and kept them at this temperature for at least 2 months. The pyloric rhythm in these animals was also regular over a wide range of temperatures (Figure 8). During cold ramps, the pyloric rhythm remained regular until a temperature of 2.69 ± 0.8°C (*N* = 12) was reached, after which it showed intermittent activity. The rhythm crashed at 1.39 ± 0.7°C (*N* = 9). In 3 animals, the final crash temperature could not be reached (see Methods). They were thus excluded from the calculation. During hot ramps, the rhythm remained regular up to 32.49 ± 2.1°C (*N* = 11). The intermittent activity condition was observed up to 37.37 ± 1.1°C (*N* = 11) where the rhythm crashed. In one of the recordings (experiment 11 in Figure 8), the recording was lost after the cold ramp and could not be recovered. This reduced the number of animals from 12 to 11 for the hot ramps. There was again substantial variability of the crash temperatures.

There was no significant difference in cold crash temperatures when the three different acclimatization groups for *C. maenas* were compared. For the temperature at which the rhythm entered the intermittent pattern during the cold ramp, the 15°C-acclimated animals from Helgoland differed significantly from their 8°C-acclimated peers. For hot ramps, more significances were observed. In both warm-acclimated groups, the 15°C-acclimated animals from Helgoland and from Vigo, the pyloric rhythm was significantly more robust against high temperatures. For both groups, the temperature at which intermittent activity began during the hot ramp, and the final crash point were significantly higher than in the 8°C-acclimated animals. Thus, in summary, the two warm-acclimated groups (15°C Helgoland and 15°C Vigo) were not significantly different from each other, but they differed significantly from the 8°C cold-acclimated animals (Supplementary Data Sheets 1 XII–XV).

## Discussion

### General observations

Our results reveal that *H. sanguineus* and *C. maenas* exhibited a reliable and consistently active pyloric pattern across a broad range of acute temperature changes. The ~30°C wide temperature range in which the rhythm of both species was regular is more extensive than in the two crab species tested previously (*C. borealis* and *C. pagurus*). In these species, hot crashes occur well below 30°C, and while the cold crash points have never been measured, animals inhabiting waters between 5°C and 14°C show behavioral responses at temperatures close to freezing (Bakke et al., 2019). This aligns well with their approximate temperature and latitude ranges, spanning from Florida to Newfoundland and 2 to 24°C in *C. borealis* (Haefner, 1977; Stehlik et al., 1991; Lewis and Ayers, 2014) and northern Norway to Spain (5°C and 21°C, respectively)^1^ for *C. pagurus* (Bakke et al., 2019). Moreover, our findings are consistent with previous investigations that determined the critical thermal maximum (CTmax) of *C. pagurus* and *C. maenas* by assessing the righting response of crabs (Cuculescu et al., 1998). Not only is the CTmax lower for *C. pagurus* compared to *C. maenas*, but the CTmax values for both species closely resembled the temperature at which the pyloric rhythm exhibited a hot crash. This was the case for *C. maenas* (as determined in our study) and for *C. pagurus* (as determined in our previous study, (Soofi et al., 2014)).

Our data also support previous studies that have characterized *C. maenas* and *H. sanguineus* as eurythermic. *C. maenas* survives temperatures from freezing to over 35°C, with intact cardiac function up to 37°C (Klassen and Locke, 2007; Tepolt and Somero, 2014). Similarly, *H. sanguineus* is known to withstand temperatures between 0°C and 28°C in its native range from Sakhalin Island (Russia) to Taiwan (Klassen, 2012). We found a large neuronal temperature robustness in both species that exceeds the temperature extremes they encounter in the habitat we collected them from (~4°C in January to ~17°C in August,^2^ with respective minimum and maximum values of ~0°C and 20°C (De Amorim et al., 2023)). The hot crash temperatures were about 10°C higher than that, suggesting that the temperature range in which the rhythm functions is large enough to maintain activity across all seasons. This large temperature robustness may act as a safety factor providing resilience against extreme temperatures, such as exposure to sun heating, occasional heatwaves, or warm ocean currents, and may act in addition to behavioral patterns that allow the animals to avoid the hottest temperatures and dampen thermal fluctuations in the intertidal by remaining under rocks or the macroalgal canopy. Alternatively, the observed robustness may not solely be attributed to temperature fluctuations but also to its ability to withstand other concurrent perturbations. Changes in temperature often coincide with variations in oxygen levels, pH, and salinity (Stein and Harzsch, 2021), and although only a few studies have investigated the effects of combined stressors (Ratliff et al., 2021), it is plausible that their presence diminishes the overall stability of neuronal activity. Therefore, the observed high temperature robustness might indicate an overall resilience against multiple perturbations. In situations where stressors coincide, this resilience may be crucial in maintaining the rhythmic activity even at cooler temperatures.

### Phase constancy

Phase constancy is a hallmark of the pyloric circuit and thought to underlie the reliable generation of the rhythmic activity associated with digestive processes. Studies across several decapod species have demonstrated that the three pyloric phases are immediately recognizable in each animal and that there is a rather small inter-animal variability in phase relationship (Bucher et al., 2005; Marder and Bucher, 2007; Stein et al., 2016). We observed the same in *H. sanguineus* and *C. maenas*, suggesting that a precise coordination of the rhythmic pyloric output also contributes to the rhythm’s functional integrity in these species. However, in contrast to previous studies (Tang et al., 2010; Soofi et al., 2014), phase relationships changed with temperature in the species studied here (Figure 6). Changes to the phase relationships have been associated with changes in neuromodulatory states and behavioral conditions (Marder and Bucher, 2007), which suggests that a reorganization of the circuit dynamics may arise from temperature-dependent changes in cellular and synaptic properties. This could serve to maintain the triphasic movements of the pylorus and optimize digestive functions under different thermal regimes. Past studies have shown that the muscles’ response to neuronal input diminishes with increasing temperature (Thuma et al., 2013), but also that food transit times increase with temperature (McGaw and Whiteley, 2012). Altering phase relationships of the innervating neurons may provide the key neuronal input necessary to allow the pyloric filter apparatus to continue to function in different thermal conditions, particularly in species inhabiting intertidal environments with much larger and more rapidly changing thermal conditions.

We also detected differences in how phase relationships and cycle frequency changed between *H. sanguineus* and *C. maenas*. In *H. sanguineus*, phase relationships changed mainly at high temperatures, when cycle frequency decreased (Figure 6). In contrast, phase relationships remained the same on the cold side, despite a decrease in cycle frequency. In *C. maenas*, phase relationships changed mostly at intermediate temperatures while the cold and warm phase relationships were similar. This was the case despite dramatic differences in cycle frequency between low and high temperatures. The phase relationships of the pyloric neurons in both species were thus not a function of cycle frequency. Instead, it is more likely that species-specific differences in the neural circuitry and intrinsic neuronal properties contribute to the observed discrepancies between *H. sanguineus* and *C. maenas*, despite sharing an ecological niche and habitat.

These species-specific differences in response profiles suggest a potential evolutionary adaptation to different thermal environments and ecological conditions. Previous studies have suggested that *H. sanguineus* is better adapted to warmer habitats than *C. maenas*. For example, the survival rate of larval *H. sanguineus* is lower at cold temperatures (Espinosa-Novo et al., 2023), and adults show better tolerance to high temperatures and thermal fluctuations than *C. maenas*, especially under food limitation (Giménez et al., 2021; Espinosa-Novo et al., 2023). The predicted northern range edge of *H. sanguineus* is also substantially south of the one currently observed for *C. maenas* (Giménez et al., 2020). Our own data show that the pyloric cycle frequency in *H. sanguineus* is generally lower (Figure 6), and especially so at the temperature extremes. The pyloric cycle frequency is the sole factor determining the speed with which the pyloric muscles contract. At 30°C, the pyloric cycle frequency was 3.5 times slower than in *C. maenas.* The energy demands on the muscle and the neuronal level must thus be much lower. Given that *H. sanguineus* also exhibits a two-fold higher respiration rate than *C. maenas* at high temperature (Jungblut et al., 2018), which provides more oxygen, it likely has an energetic advantage over *C. maenas.*

Looking at the robustness of the pyloric rhythm, we made an interesting observation: When acclimated to 15°C, the temperature robustness of the *H. sanguineus* pyloric rhythm was more like that of 8°C-acclimated *C. maenas* than that of 15°C-acclimated *C. maenas*. For example, hot crashes in 15°C-acclimated *H. sanguineus* were similar to 8°C-acclimated *C. maenas*, but they occurred at lower temperatures than in 15°C-acclimated *C. maenas*. On the other hand, the hot crash temperature of 25°C-acclimated *H. sanguineus* were similar to the 15°C-acclimated *C. maenas*. This suggests that when both species occupy the same temperature habitat, adult *C. maenas* show a better robustness against acutely increasing temperatures. However, the high crash temperature of 15°C-acclimated *C. maenas* (>37°C) is already close to the critical thermal maximum, suggesting that additional warm acclimatization cannot elevate the hot crash temperature any further.

### Temperature acclimation

Decapod crustaceans are well-known to acclimate to new habitat temperatures (Somero, 2010; Somero, 2012; McGaw and Curtis, 2013; Tepolt and Somero, 2014). For example, in *C. maenas*, the CTmax of the righting response shifts from 31.8°C to 35.4°C when the animals are acclimating from 8°C to 22°C (Cuculescu et al., 1998). The neuronal side of acclimatization is far less clear. Two competing hypotheses have been proposed. In the first, the nervous system shows similar acclimatization responses as the behavioral performance. In the second, the nervous system’s temperature robustness is large enough to not require acclimatization and changes to survival and behavioral performance are due to effects on other, non-neuronal tissues. Our data support the first hypothesis, because the range of regular pyloric activity moved after acclimation in both species. For *C. maenas*, the observed hot crash temperatures are even similar to those measured for behavioral performance (Cuculescu et al., 1998).

Previous studies had suggested that the pyloric system acclimates to different seasons (Marder and Rue, 2021). However, these studies were correlational and carried out over several years. It thus remained unclear if the observed correlation between pyloric temperature responses and environmental temperatures were by distinct genetic or evolutionary backgrounds of different crab populations sampled. We used the same pool of crabs for the control and acclimated condition, eliminating this uncertainty.

Acclimatization of neuronal responses could be attributed to several underlying mechanisms, including the regulation of voltage-gated ion channels. Stomatogastric neurons possess various ion channels whose currents and gating variables show large and distinct sensitivities to temperature (Tang et al., 2010). Modeling studies have suggested that the correlated expression of ion channels can lead to temperature robustness even if their temperature sensitivities are distinct, and that many sets of conductances can achieve temperature robustness (O’Leary and Marder, 2016; DeMaegd and Stein, 2020). Long-term, targeted changes in the composition of existing ion channels may thus contribute acclimatization. Such changes can come about through many different mechanisms, including the differential expression of ion channels, RNA editing of existing channels, the expression of different isoforms or splice variants (Johnson et al., 2011; Lin and Baines, 2015; Schulz and Lane, 2017), and the modulation of ionic conductances by neuromodulators (Städele et al., 2015; DeMaegd and Stein, 2021; Stadele and Stein, 2022).

### Degeneracy of neuronal crash behaviors.

Our data reveal a significant variability in the crash temperature and the crash behaviors (the activity types produced) between individuals in each species (Figure 7; Supplementary Data Sheets 3, 4). These findings corroborate previous studies in other species (Marder and Rue, 2021) and are thought to be a consequence of degenerate circuits, i.e., the fact that there is a 2- to 6-fold variability in the expression of ion channel genes and their respective ionic currents within single neuron types (Schulz et al., 2006; Khorkova and Golowasch, 2007; Schulz et al., 2007; Goaillard et al., 2009; Ransdell et al., 2013; Northcutt et al., 2019) while circuit output (the pyloric rhythm) is almost indistinguishable between individuals (Bucher et al., 2005). One of the consequences of this variability is that individuals show varying responses to perturbations (Ratliff et al., 2021). In our case, the varying crash temperatures suggest that both species, *H. sanguineus* and *C. maenas*, possess substantial inter-animal variability in their pyloric circuit parameters.

We noticed that cold crash variability was consistently smaller than hot crash variability (Figure 7). Most cold crashes can be described as a slowing down of the rhythm, followed by loss of one neuron type (usually LP) and then a loss of rhythmicity when the PD neurons fail. In contrast, no consistent description is possible for hot crashes because of the various activities observed. Given that the underlying variability in neuronal properties is the same during cold and hot crashes, these observations suggest that the system is less perturbed by cold temperatures than by hot temperatures. Therefore, the cryptic variability remains mostly hidden at cold temperatures. We also noticed that the variability of the crash temperatures changed after acclimation. Hot acclimation reduced the variability of the hot crash temperatures by a factor of two. Two non-exclusive explanations for this phenomenon are likely. First, the crash temperatures after acclimation are now closer to the upper thermal limit that the system can reach. In this case, the reduced variability after acclimation is simply due to the fact that the upper thermal limit truncates the potential to observe variability, because the rhythm cannot continue beyond this temperature. A more interesting second scenario is related to the number of possible combinations of neuronal properties that can sustain a functional activity: cryptic variability of ion channel gene expression cannot be random (Caplan et al., 2014). Instead, there is a limited number of neuron type-specific correlations that produce the stereotypical pyloric rhythm (Khorkova and Golowasch, 2007; Temporal et al., 2012; O’Leary et al., 2013; Golowasch, 2019). That number should also depend on which perturbations the circuit has to be resilient against. Higher temperatures, like after warm-acclimation, challenge the circuit more than lower temperatures, resulting in fewer adequate solutions in parameters space the circuit can select from than at lower acclimation temperatures. In other words, fewer combinations of intrinsic properties are available to maintain the rhythm, leading to a more limited repertoire of intrinsic properties across individuals. This could then result in the smaller inter-animal variability of the hot crash temperatures that we observed. In the extreme case of acclimatization to temperatures close to the upper thermal limit, no additional combinations may be available, making it impossible to acclimate to even higher temperatures (Somero, 2010; Tepolt and Somero, 2014).

There is further support for this idea in *C. maenas*, the total range of regular activity increased after acclimation (Figure 7B). A larger range represents a greater challenge and should limit the number of available combinations even further than the mere shift toward higher temperature after acclimation we observed in *H. sanguineus*. This predicts that inter-animal variability should be reduced more strongly in *C. maenas*. Our data are consistent with this idea, showing that cold crash temperature variability diminished in *C. maenas*, but not in *H. sanguineus*.

Degeneracy is a key characteristic of complex biological systems and has been shown to exist in several other neuronal systems (Golowasch, 2014; Goaillard and Marder, 2021). In the context of temperature fluctuations, degeneracy allows for variations in cellular and synaptic properties in individuals of a species, accommodating different thermal sensitivities and maintaining the overall resilience of the species against temperature changes. An acclimatization-induced reduction of degeneracy-driven resilience may challenge the survival and performance of crab species in their natural habitats, in particular when they experience rapid and wide-ranging temperature changes.

## Data availability statement

The original contributions presented in the study are included in the article/Supplementary material, further inquiries can be directed to the corresponding author.

## Author contributions

WS: Conceptualization, Data curation, Formal Analysis, Funding acquisition, Investigation, Methodology, Project administration, Resources, Supervision, Validation, Visualization, Writing – original draft, Writing – review & editing. GT: Methodology, Resources, Writing – review & editing. LG: Methodology, Resources, Writing – review & editing. NE-N: Methodology, Resources, Writing – review & editing. JPG: Methodology, Resources, Writing – review & editing. AV-G: Conceptualization, Validation, Writing – review & editing. SH: Conceptualization, Funding acquisition, Methodology, Project administration, Resources, Supervision, Validation, Writing – review & editing.

## Footnotes

^1^https://seatemperature.info

^2^https://seatemperature.info/helgoland-water-temperature.html
