# Supplementary material for: Thermal acclimation and habitat-dependent differences in temperature robustness of a crustacean motor circuit
Source: Front Cell Neurosci. 2023 Oct 18;17:1263591. doi: 10.3389/fncel.2023.1263591 (PMC10619761; doi:10.3389/fncel.2023.1263591)
Supplement: Supplementary file 1 [file Data_Sheet_1.pdf]

Supplemental 1. Statistical analyses carried out.

Intermittent to hot crash temperature	*Carcinus maenas* Mean [°C]	*Carcinus maenas* SD [°C]	*Carcinus maenas* N	*Hemigrapsus sanguineus* Mean [°C]	*Hemigrapsus sanguineus* SD [°C]	*Hemigrapsus sanguineus* N	T-test P value
6°C	0.509506	0.206023	7	0.305397	0.060786	9	P=0.013
10°C	0.613633	0.181435	7	0.449368	0.061101	9	P=0.023
14°C	0.565361	0	1	0.634347	0.114689	9	ns
18°C	0.763826	0.298372	7	0.735983	0.351913	8	ns
22°C	0.891471	0.528896	7	0.732817	0.420991	9	ns
26°C	1.323203	0.369619	7	0.663857	0.462223	10	P=0.007
30°C	1.964928	0.443542	4	0.549438	0.373088	6	P<0.001

I. Pyloric cycle frequency of *Hemigrapsus sanguineus* and *Carcinus maenas*, averaged, at different temperatures. T-tests between the two species were carried out separately for each temperature.

Intermittent to hot crash temperature	Mean [°C]	N	SD [°C]	5°C	10°C	21°C	28°C
5°C	0.26942	9	0.032235				X
10°C	0.239917	9	0.039263				X
21°C	0.21654	9	0.068605				
28°C	0.169129	9	0.086104	X	X		

II. End of activity phase of PD in *Hemigrapsus sanguineus*, averaged, at different temperatures. One Way Analysis of Variance, $F(3,32)=4.394$, $P<0.05$ SNK post-hoc test. There was an overall significant difference ($P<0.001$). Significant differences of the pairwise comparisons are marked with “X” ($P<0.05$).

Intermittent to hot crash temperature	Mean [°C]	N	SD [°C]	5°C	10°C	21°C	28°C
5°C	0.464865	9	0.056863			X	X
10°C	0.413869	9	0.036238			X	X
21°C	0.323752	9	0.08163	X	X		X
28°C	0.20958	9	0.097434	X	X	X	

III. Start of activity phase of LP in *Hemigrapsus sanguineus*, averaged, at different temperatures. One Way Analysis of Variance, $F(3,32)= 21.819$, $P<0.05$ SNK post-hoc test. There was an overall significant difference ($P <0.001$). Significant differences of the pairwise comparisons are marked with "X" ($P<0.05$).

Intermittent to hot crash temperature	Mean [°C]	N	SD [°C]	5°C	10°C	21°C	28°C
5°C	0.639205	9	0.09285			X	X
10°C	0.608965	9	0.052151			X	X
21°C	0.482641	9	0.089899	X	X		X
28°C	0.301311	9	0.113375	X	X	X	

IV. End of activity phase of LP in *Hemigrapsus sanguineus*, averaged, at different temperatures. One Way Analysis of Variance, $F(3,32)= 26.312$, $P<0.05$ SNK post-hoc test. There was an overall significant difference ($P <0.001$). Significant differences of the pairwise comparisons are marked with "X" ($P<0.05$).

Intermittent to hot crash temperature	Mean [°C]	N	SD [°C]	5°C	10°C	21°C	28°C
5°C	0.673621	9	0.107846			X	X
10°C	0.633993	9	0.055865			X	X
21°C	0.53054	9	0.087727	X	X		X
28°C	0.333978	9	0.114293	X	X	X	

V. Start of activity phase of PY in *Hemigrapsus sanguineus*, averaged, at different temperatures. One Way Analysis of Variance, $F(3,32) = 23.380$, $P<0.05$ SNK post-hoc test. There was an overall significant difference ($P < 0.001$). Significant differences of the pairwise comparisons are marked with “X” ($P<0.05$).

Intermittent to hot crash temperature	Mean [°C]	N	SD [°C]	5°C	10°C	21°C	28°C
5°C	0.979496	9	0.020094				
10°C	0.984859	9	0.006706				
21°C	0.996709	9	0.033742				
28°C	0.987044	9	0.005711				

VI. End of activity phase of PY in *Hemigrapsus sanguineus*, averaged, at different temperatures. One Way Analysis of Variance, $F(3,32) = 1.149$. There was no statistically significant difference ($P = 0.344$).

Intermittent to hot crash temperature	Mean [°C]	N	SD [°C]	5°C	10°C	21°C	28°C
5°C	0.26942	9	0.032235			X	
10°C	0.239917	9	0.039263			X	
21°C	0.21654	9	0.068605	X	X		X
28°C	0.169129	9	0.086104			X	

VII. End of activity phase of PD in *Carcinus maenas*, averaged, at different temperatures. Friedman Repeated Measures Analysis of Variance on Ranks. $\chi^2 = 12.333$, df = 3, P = 0.006. P<0.05 SNK post-hoc test. Significant differences of the pairwise comparisons are marked with "X" (P<0.05).

Intermittent to hot crash temperature	Mean [°C]	N	SD [°C]	5°C	15°C	21°C	31°C
5°C	0.464865	9	0.056863		X	X	X
15°C	0.413869	9	0.036238	X			
21°C	0.323752	9	0.08163	X			
31°C	0.20958	9	0.097434	X			

VIII. Start of activity phase of LP in *Carcinus maenas*, averaged, at different temperatures. One Way RM Analysis of Variance, $F(3,24) = 12.240$, P<0.05 SNK post-hoc test. There was an overall significant difference (P <0.001). Significant differences of the pairwise comparisons are marked with "X" (P<0.05).

Intermittent to hot crash temperature	Mean [°C]	N	SD [°C]	5°C	15°C	21°C	31°C
5°C	0.464865	9	0.056863		X	X	X
15°C	0.413869	9	0.036238	X		X	
21°C	0.323752	9	0.08163	X	X		X
31°C	0.20958	9	0.097434	X		X	

IX. End of activity phase of LP in *Carcinus maenas*, averaged, at different temperatures. One Way RM Analysis of Variance, $F(3,24)= 15.146$, $P<0.05$ SNK post-hoc test. There was an overall significant difference ($P <0.001$). Significant differences of the pairwise comparisons are marked with “X” ($P<0.05$).

Intermittent to hot crash temperature	Mean [°C]	N	SD [°C]	5°C	15°C	21°C	31°C
5°C	0.464865	9	0.056863			X	
15°C	0.413869	9	0.036238			X	
21°C	0.323752	9	0.08163	X	X		X
31°C	0.20958	9	0.097434			X	

X. Start of activity phase of PY in *Carcinus maenas*, averaged, at different temperatures. One Way RM Analysis of Variance, $F(3,24)= 14.705$, $P<0.05$ SNK post-hoc test. There was an overall significant difference ($P <0.001$). Significant differences of the pairwise comparisons are marked with “X” ($P<0.05$).

Intermittent to hot crash temperature	Mean [°C]	N	SD [°C]	5°C	15°C	21°C	31°C
5°C	0.464865	9	0.056863				
15°C	0.413869	9	0.036238				
21°C	0.323752	9	0.08163				
31°C	0.20958	9	0.097434				

XI. End of activity phase of PY in *Carcinus maenas*, averaged, at different temperatures. One Way RM Analysis of Variance, $F(3,24)= 1.812$. There was no statistically significant difference ($P = 0.172$).

Intermittent to cold crash temperature	Mean [°C]	N	SD [°C]	*H. sanguineus* 15°C	*H. sanguineus* 25°C	*C. maenas* 8°C Helgoland	*C. maenas* 15°C Helgoland	*C. maenas* 15°C Vigo
H. sanguineus 15°C	3.97	8	1.054419		X			
H. sanguineus 25°C	1.64	7	0.766085	X		X	X	X
C. maenas *8*°C	1.162	10	1.446719		X			
C. maenas 15°C Helgoland	1.805556	9	0.533565		X			
C. maenas 15°C Vigo	1.388889	9	0.662947		X			

XII. Averaged temperatures at which the pyloric rhythm of the different groups encountered a cold crash. One Way Analysis of Variance, $F(4,38)=11.248$, $P<0.05$ SNK post-hoc test. There was an overall significant difference ($P <0.001$). Significant differences of the pairwise comparisons are marked with “X” ($P<0.05$).

Stable to cold intermittent temperature	Mean [°C]	N	SD [°C]	*H. sanguineus* 15°C	*H. sanguineus* 25°C	*C. maenas* 8°C Helgoland	*C. maenas* 15°C Helgoland	*C. maenas* 15°C Vigo
H. sanguineus 15°C	4.6375	8	0.9068		X			
H. sanguineus 25°C	2.735	8	1.0028	X		X		X
C. maenas 8°C	2.417	10	2.0719		X			
C. maenas 15°C Helgoland	3.73	9	0.521					
C. maenas 15°C Vigo	2.6875	12	0.7667		X			

XIII. Averaged temperatures at which the pyloric rhythm of the different groups lost stable activity and became intermittent during cold ramps. One Way Analysis of Variance, $F(4,42)= 5.249$, $P<0.05$ SNK post-hoc test. There was an overall significant difference ($P =0.002$). Significant differences of the pairwise comparisons are marked with “X” ($P<0.05$).

Stable to hot intermittent temperature	Mean [°C]	N	SD [°C]	*H. sanguineus* 15°C	*H. sanguineus* 25°C	*C. maenas* 8°C Helgoland	*C. maenas* 15°C Helgoland	*C. maenas* 15°C Vigo
H. sanguineus 15°C	35.275	8	1.5998		X		X	X
H. sanguineus 25°C	30.46	10	2.907	X		X	X	X
C. maenas *8*°C	30.07	10	1.8969		X		X	X
C. maenas 15°C Helgoland	33.2944	9	0.8539	X	X	X		
C. maenas 15°C Vigo	32.4855	11	2.0637	X	X	X		

XIV. Averaged temperatures at which the pyloric rhythm of the different groups lost stable activity and became intermittent during hot ramps. One Way Analysis of Variance, $F(4,43)= 10.022$, $P<0.05$ SNK post-hoc test. There was an overall significant difference ($P <0.001$). Significant differences of the pairwise comparisons are marked with "X" ($P<0.05$).

Intermittent to hot crash temperature	Mean [°C]	N	SD [°C]	*H. sanguineus* 15°C	*H. sanguineus* 25°C	*C. maenas* 8°C Helgoland	*C. maenas* 15°C Helgoland	*C. maenas* 15°C Vigo
H. sanguineus 15°C	35.275	8	1.5998		X		X	X
H. sanguineus 25°C	30.46	10	2.907	X		X		
C. maenas 8°C	30.07	10	1.8969		X		X	X
C. maenas 15°C Helgoland	33.2944	9	0.8539	X		X		
C. maenas 15°C Vigo	32.4855	11	2.0637	X		X		

XV. Averaged temperatures at which the pyloric rhythm of the different groups encountered a hot crash. One Way Analysis of Variance, $F(4,43)= 15.898$, $P<0.05$ SNK post-hoc test. There was an overall significant difference ($P <0.001$). Significant differences of the pairwise comparisons are marked with "X" ($P<0.05$).
