# Supplementary material for: Thermal acclimation and habitat-dependent differences in temperature robustness of a crustacean motor circuit
Source: Front Cell Neurosci. 2023 Oct 18;17:1263591. doi: 10.3389/fncel.2023.1263591 (PMC10619761; doi:10.3389/fncel.2023.1263591)

Supplemental 2. We observed spontaneous gastric mill rhythms in a subset of the recorded animals. Top: Recordings of the *lgn, mvn,* and *lvn,* in *H. sanguineus.* The rhythmic activity of the lateral gastric neuron (LG) can be seen on the *lgn.* The gastropyloric neurons IC (inferior cardiac) and VD (ventricular dilator) on the *mvn* are modulated in time with the LG bursts, as described in other crab species (Nusbaum and Beenhakker, 2002). Bottom: Recordings of the *lgn, pdn,* and *lvn,* in *C. maenas.* The rhythmic activity of the lateral gastric neuron (LG) can be seen on the *lgn.*

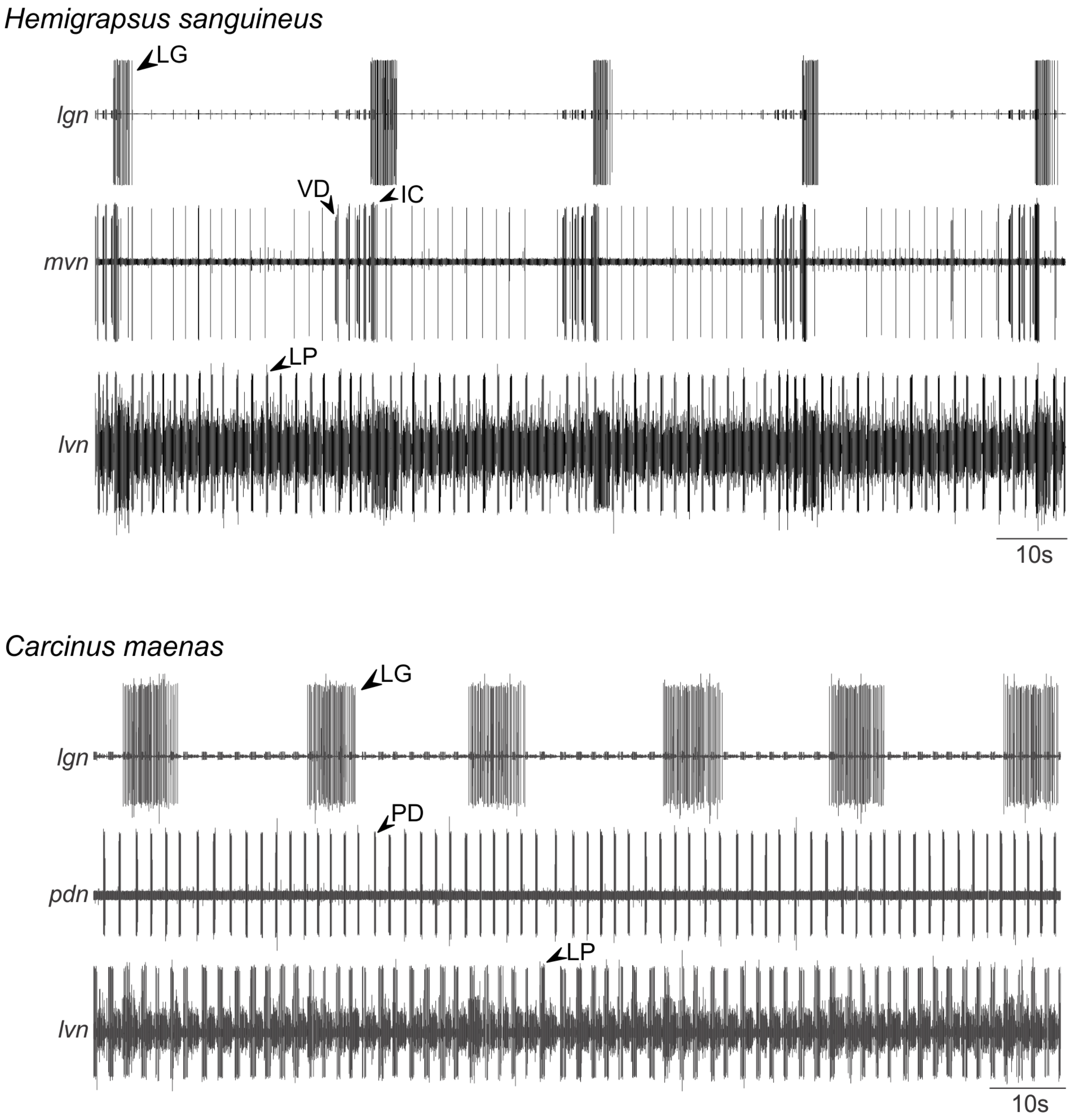

Supplement: Supplementary file 2 [file Data_Sheet_2.pdf]
