# Supplementary material for: Thermal acclimation and habitat-dependent differences in temperature robustness of a crustacean motor circuit
Source: Front Cell Neurosci. 2023 Oct 18;17:1263591. doi: 10.3389/fncel.2023.1263591 (PMC10619761; doi:10.3389/fncel.2023.1263591)

Supplemental 3. During a heat crash, the activity of the pyloric neurons varied strongly between animals of the same species. Recordings of the *lvn* from three different *H. sanguineus* are shown. Left: regular pyloric rhythm before the crash, showing the usual three pyloric phases. Right: activity during the crash. Note the difference between animals.

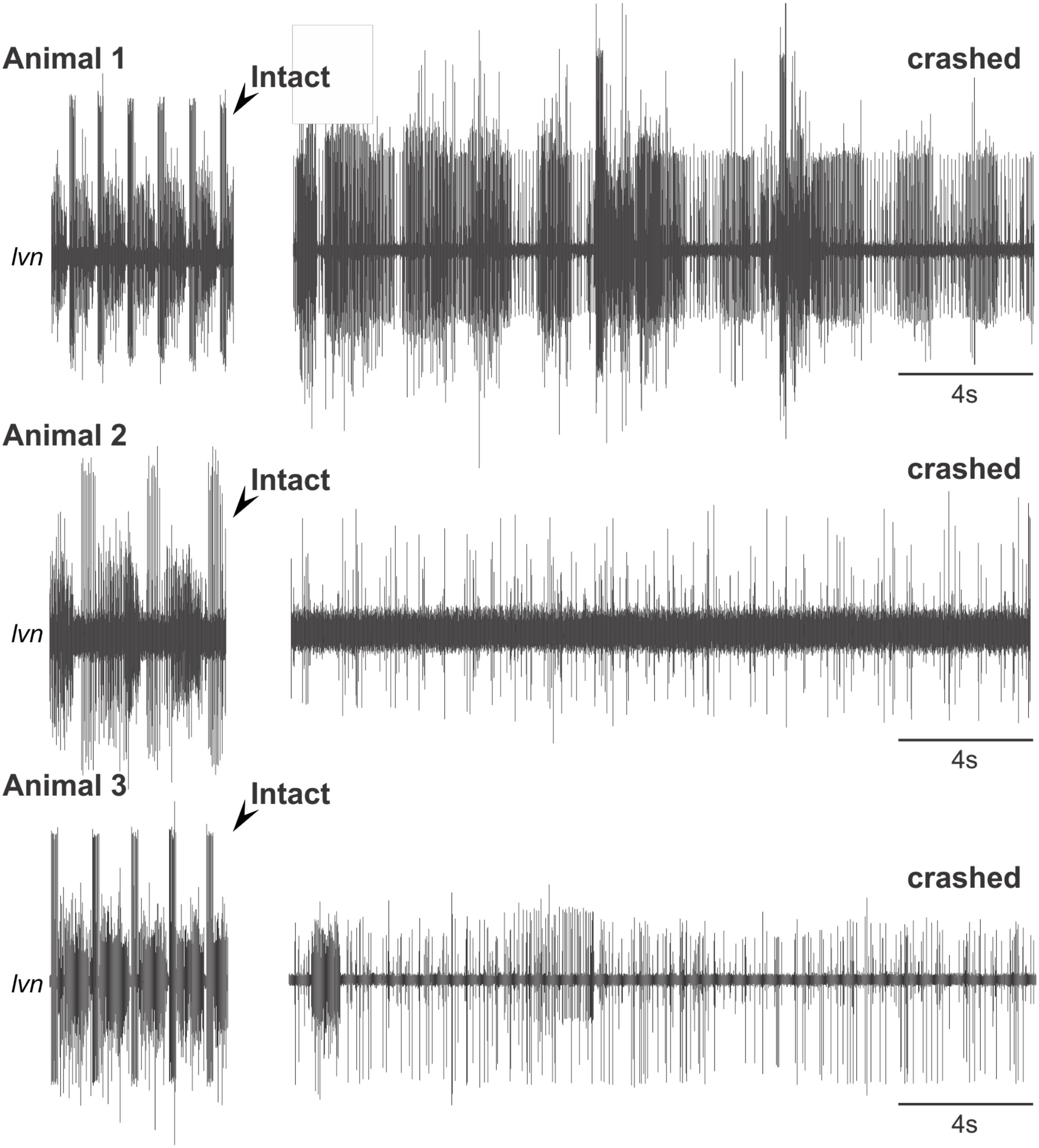

Supplement: Supplementary file 3 [file Data_Sheet_3.pdf]
