# Supplementary material for: Thermal acclimation and habitat-dependent differences in temperature robustness of a crustacean motor circuit
Source: Front Cell Neurosci. 2023 Oct 18;17:1263591. doi: 10.3389/fncel.2023.1263591 (PMC10619761; doi:10.3389/fncel.2023.1263591)

Supplemental 5. The direction of the temperature ramps had little effect on the crash temperatures. Summary diagrams of pyloric activity over temperature of three *H. sanguineus*. Ruby: Rhythmicity was absent ('crash'). Orange: Intermittent activity with one neuron type failing to fire or with intermittent rhythmicity. Teal: regular rhythmic activity with constant phase relationships. The outer boundaries of the ruby zones mark the minimum and maximum temperatures tested. Top row of each animal: temperature was first increased (hot ramp, red arrow) and then decreased (cold ramp, blue arrow). Bottom row of each animal: temperature was first decreased (cold ramp, blue arrow) and then increased (hot ramp, red arrow).

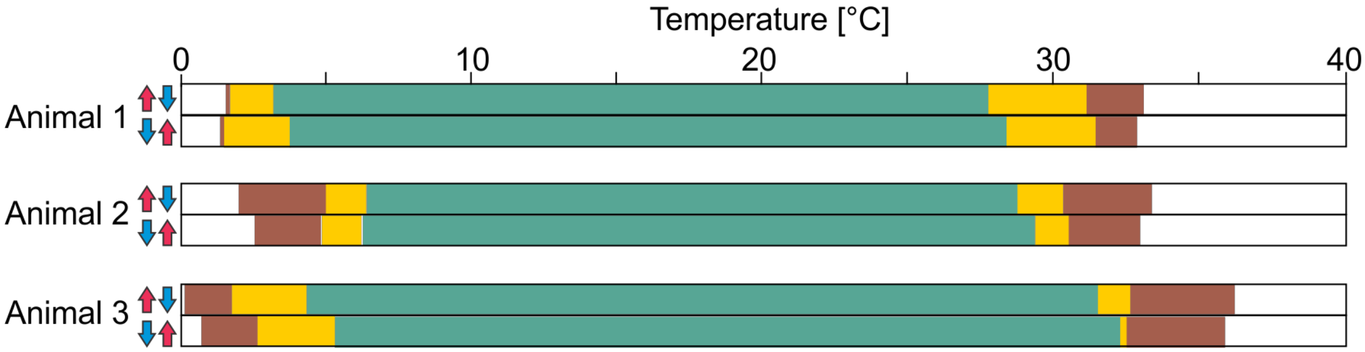

Supplement: Supplementary file 5 [file Data_Sheet_5.pdf]
